# AAA Research Methodology I: Overcoming Linguistic and Cultural Barriers in Aging Research Among Asians

**DOI:** 10.1093/geroni/igaa057.2952

**Published:** 2020-12-16

**Authors:** Wenjun Li, Su-I Hou

## Abstract

Asians are the largest and the fastest growing segment of the world population. Asian immigrants are the second largest immigrant population in the U.S. However, their age-related social and health issues are understudied. Because studies on older Asians are often scattered geographically and small-sized and study instruments are usually inconsistent in context, language and culture, it is difficult to synthesize findings from different studies on Asians. Little data exist to support health promotion, policy evaluation and clinical practice in this population. To advance research into aging among Asians, a fundamental step is to create content-relevant, linguistically and culturally appropriate research instruments, and encourage use of these consistent and comparable instruments across studies. This symposium brings together four abstracts that report the development and adaptation of linguistically and culturally appropriate survey instruments for health and behavioral studies in older Asians. The topics range from development of new scales for generative concern and acts in older Singaporeans, assessment of appropriateness of health literacy measurements among East Asian older adults, validation of Health Aging Instrument in Southeastern Asian older adults. Based on the experiences in these studies, the group will discuss the need and strategies to develop an international network to promote resource sharing and research collaborations across geographic boundaries and disciplinary divisions. By bringing together the isolated but talented Asian health researchers, the new network may accelerate the growth of research on Aging Among Asians. This symposium is a collaborative effort of the Aging Among Asians Interest Group.

